# Machine Learning and Deep Learning for Healthcare Data Processing and Analyzing: Towards Data-Driven Decision-Making and Precise Medicine

**DOI:** 10.3390/diagnostics15081051

**Published:** 2025-04-21

**Authors:** Haipeng Liu, Rajesh Kumar Tripathy

**Affiliations:** 1 Centre for Intelligent Healthcare, Coventry University, Coventry CV1 5RW, UK; 2 Department of Electrical and Electronics Engineering, BITS-Pilani, Hyderabad Campus, Hyderabad 500078, India

Artificial intelligence (AI) is reshaping the landscape of healthcare data. Alongside electronic medical records (EHRs), AI algorithms are accelerating the collection, management, processing, and analysis of healthcare data [1]. Its power was recently showcased through the realization of AI-based methods for the early detection and fine-grained severity evaluation of COVID-19. Machine learning and deep learning models enable the automatic processing of multimodal healthcare data, including EHRs, physiological signals, and medical images. In this Special Issue, “Machine Learning and Deep Learning for Healthcare Data Processing and Analyzing”, we collect eleven research and opinion articles focusing on the application of AI models to different types of healthcare data. The published studies in this Special Issue provide up-to-date examples of AI uses in a healthcare context (Table 1).

Machine learning can comprehensively analyze different features and estimate the risk, enhancing diagnoses and prognoses. Based on a longitudinal dataset of 150 people, Alshamlan et al. compared some common machine learning models, including support vector machine (SVM), random forest (RF), and logistic regression (LR) approaches, in predicting Alzheimer’s disease. Minimum redundancy maximum relevance (mRMR) and mutual information (MI) were employed for feature selection, and LR combined with mRMR achieved the highest accuracy of 99.08% in predicting Alzheimer’s disease. Their results highlighted the role of AI in disease prediction and clinical decision-making.

Toader et al. used machine learning models to predict clinical outcomes in microsurgical clipping treatments of cerebral aneurysms, based on a dataset of 344 patients’ preoperative characteristics. Validating prediction outcomes on the Glasgow Outcome Scale (GOS), their extreme gradient boosting (XGB) model outperformed the others and achieved an area under the receiver operating characteristic curve (AUC ROC) of 0.72 ± 0.03 for specific GOS outcome prediction, and an AUC ROC of 0.78 ± 0.02 for the binary classification of outcomes. These results demonstrate the potential of machine learning as a tool for predicting the surgical outcomes of ruptured cerebral aneurysm treatments. Moreover, the study underscores the need for high-quality, large-scale datasets and external validation in order to enhance the reliability and generalizability of machine learning models.

Deep learning enables the in-depth analysis of clinical images, making automatic classification and fine-grained feature analysis possible. In their contribution, Hadj-Alouane et al. propose an AI framework for the diagnosis and severity classification of Parkinson’s Disease using video data captured in uncontrolled environments. Deep learning models, including a convolutional neural network (CNN), residual network (ResNet), and vision transformer (ViT), were deployed in gait analysis based on skeleton energy images (SEIs) of gait sequences. Although only 167 subjects were included, 90% accuracy was achieved. Their proposed framework provides a new potential pathway for the cost-effective early detection of Parkinson’s Disease in normal healthcare settings.

Mudavadkar et al. used ensemble models that mixed different deep learning architectures to improve the performance of AI in detecting gastric cancer. The models were trained and tested on a database of 600 pictures of stomach cancer pathology, with the pictures cropped to different sizes. With their proposed ensemble model achieving over 90% accuracy at different resolutions, the authors concluded that ensemble models may be able to accurately detect some pathological features from smaller picture patches, empowering pathologists to diagnose gastric cancer at an early stage.

The quantification of interested features is important in diagnostics. With this in mind, Nair et al. applied AI-based quantitative lung texture analysis to high-resolution computed tomography (HRCT) images of 45 patients in order to detect subtle lung parenchymal involvement in bronchiectasis. Among the patterns detected by AI, hyperlucency was suggestive of air-trapping and alveolar destruction, while ground-glass opacity suggested active alveolitis and early interstitial inflammation. The results of this pilot study showed that AI is able to identify some minor features from HRCT that are often neglected in human-led visual assessments, offering new insights into the pathophysiology of bronchiectasis and other lung diseases.

Guo et al. developed two-dimensional (2D) and three-dimensional (3D) no new U-Net (nnU-Net) networks to segment contrast-enhanced computed tomography (CT) images and quantify the morphological features required to detect Type A aortic dissection. Of the two networks, their 3D nnU-Net architectures displayed a better performance on the CT datasets of 24 patients. While further large-scale validation is needed, the nnU-Net architectures showed the potential of the automatic segmentation and quantification of aortic structures for rapid diagnosis, surgical planning, and the subsequent biomechanical simulation of the aorta.

Bendella et al. proposed AI-based MRI brain volumetrics to distinguish between patients with idiopathic normal pressure hydrocephalus (iNPH), Alzheimer’s disease, and age- and sex-matched healthy controls by evaluating cortical, subcortical, and ventricular volumes. The study was conducted retrospectively on 123 age- and sex-matched subjects, with 41 in each group. The authors found that iNPH patients exhibited ventricular enlargement and changes in gray and white matter compared to the other two groups, with the most significant differences observed in total ventricular volume (+67%) and the lateral (+68%), third (+38%), and fourth (+31%) ventricles compared to the controls. Their AI-based MRI volumetry approach provided the quantitative evidence used to investigate the pathology of iNPH and improve patient management.

Deep learning can be used for processing physiological signals and multimodal data. Chin et al. introduced a neural network model for continuously estimating patient respiratory rate from photoplethysmogram signals with a reduced window size and lower processing requirements. The algorithm was validated on two datasets, and the best-performing model achieved a mean absolute error of 2 breaths/min at a window size of 7 s. This study demonstrates the superior performance of an AI model with a smaller window size, showing the potential for the quick, AI-based evaluation of the respiratory system.

Data preprocessing is important for optimizing the performance of AI models. Manir and Deshpande used different machine learning models to investigate the relationship between the risk factors of breast cancer incidence and survivability. Performing resampling and principal component analysis on the training dataset to enhance the performance of the classifiers, three breast cancer datasets were examined using a variety of preprocessing approaches and classification models. The best accuracies achieved were above 85% on all the datasets. The authors observed that resampling can worsen the accuracy scores of the test data, even when the training data accuracy is increased. Their results emphasize the significance of individualized approaches in the management and treatment of breast cancer, showcasing the versatility of machine learning in incorporating phenotypic variations and recognizing the heterogeneity of a disease when estimating clinical risk.

Despite these emerging AI models, there is a great need for specified AI tools for clinical use. Lohaj et al. developed a Shiny dashboard application named DESSFOCA (Decision Support System For Cardiologists) using the R language, structured around three core functionalities: discovering association rules, applying clustering methods, and identifying association rules within predefined clusters. The application was tested based on the feedback of end users. Based on the evaluation results, the authors proposed recommendations to further improve usability, such as adding a user manual and improving error messages to provide efficient feedback.

Badahman et al. compared the performance of a piece of AI-enhanced web-based software incorporated into a clinical decision support system (CDSS) with that of magnetic resonance imaging (MRI) in predicting lumbar disk herniation in a cross-sectional study of 100 patients. The software showed significant diagnostic accuracy, with an AUC ROC of 0.84, a sensitivity of 88%, and a specificity of 80%. The AI-enhanced CDSS may achieve comparable performance with MRI, which will largely reduce how time-consuming and costly the screening of patients with lumbar disk herniation is.

Although machine learning can offer efficient, automatic, and personalized diagnosis, thereby providing support for clinical decision-making, there are unmet challenges in terms of data, algorithms, and their application. Pinton compared the performance of two models in predicting the efficacy of biologic agents in ulcerative colitis. The author suggested that machine learning models based on multiple pathways, multiple ethnicities, and real-world and clinical trial data are required for data-driven decision-making and precision medicine. The author also pointed out that data quality and quantity, overfitting, generalization, and interpretability are major limitations in many machine learning models.

In summary, this Special Issue covers the major applications of AI in healthcare data analysis, including data-driven risk evaluation, medical image analysis, the quantification of image features, and physiological signal analysis (Figure 1). Besides its applications in data processing and analysis, AI-enhanced CDSSs improve clinical practice regarding decision-making, care delivery, and treatment selection, offering higher diagnostic accuracy and lower risks of medical error [2,3]. In addition to the technological challenges, there are ethical concerns surrounding AI use, specifically regarding data security issues, underrepresented minorities, and data-access disparities [4].

It should be noted that there are still unmet challenges in terms of the availability high-quality data, which limits large-scale validation [5,6]; the lack of standardized and efficient data preprocessing [7]; and the low explainability of AI approaches, which is a main obstacle towards their real-world clinical application [8]. In future research, the collection of high-quality data, the development of advanced data preprocessing frameworks (e.g., edge computing for big data [9]), and explainable AI techniques (e.g., ‘spread of relevance’ at the layer level of neural networks [10]) will accelerate the integration of AI models into medical systems [11], further reshaping the landscape of modern healthcare towards data-driven decision-making and precise medicine.

We appreciate the contributors to this Special Issue.

## Figures and Tables

**Figure 1:**
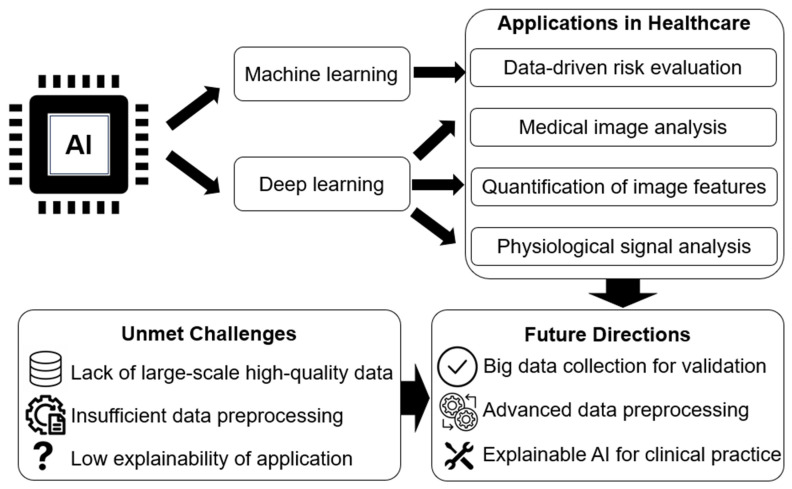
Applications of machine learning and deep learning in modern healthcare, unmet challenges, and future directions.

**Table 1:** Overview of 12 studies on artificial intelligence in healthcare.

Theme	Authors and DOI	Study Title	Key Findings
Machine learning for clinical data analysis	Alshamlan et al., 10.3390/diagnostics14192237	Improving Alzheimer’s Disease Prediction with Different Machine Learning Approaches and Feature Selection Techniques	Machine learning may offer more accurate disease prognosis for clinical decision-making.
Machine learning for clinical data analysis	Toader et al., 10.3390/diagnostics14192156	Machine Learning-Based Prediction of Clinical Outcomes in Microsurgical Clipping Treatments of Cerebral Aneurysms	High-quality large-scale datasets and external validation are essential to enhance model reliability and generalizability.
Deep learning for medical image analysis	Hadj-Alouane et al., 10.3390/diagnostics14232685	Severity Classification of Parkinson’s Disease via Synthesis of Energy Skeleton Images from Videos Produced in Uncontrolled Environments	Deep learning may enable the cost-effective early detection of Parkinson’s Disease in various healthcare settings.
Deep learning for medical image analysis	Mudavadkar et al., 10.3390/diagnostics14161746	Gastric Cancer Detection with Ensemble Learning on Digital Pathology: Use Case of Gastric Cancer on GasHisSDB Dataset	Ensemble deep learning may detect some pathological features from smaller picture patches, enabling the early diagnosis of gastric cancer.
Quantification of image features	Nair et al., 10.3390/diagnostics14242883	Artificial Intelligence Unveils the Unseen: Mapping Novel Lung Patterns in Bronchiectasis via Texture Analysis	AI-based quantified lung texture analysis provides valuable insights into the diagnosis of bronchiectasis and other lung diseases.
Quantification of image features	Guo et al., 10.3390/diagnostics14131332	Automatic Segmentation of Type A Aortic Dissection on Computed Tomography Images Using Deep Learning Approach	nnU-Net architectures enable the automatic segmentation and quantification of aorta for rapid diagnosis, surgical planning, and biomechanical simulation.
Quantification of image features	Bendella et al., 10.3390/diagnostics14131422	Brain and Ventricle Volume Alterations in Idiopathic Normal Pressure Hydrocephalus Determined by Artificial Intelligence-Based MRI Volumetry	Integrating AI volumetry with traditional radiologic measures can reveal new pathological features involving the supratentorial white matter, aiding in the identification of iNPH and patient management.
Deep learning for physiological signal analysis	Chin et al. 10.3390/diagnostics14030284	A Novel Respiratory Rate Estimation Algorithm from Photoplethysmogram Using Deep Learning Model	Deep learning can estimate respiratory rate from photoplethysmography signals with short window sizes for continuous monitoring.
Data preprocessing in machine learning	Manir and Deshpande, 10.3390/diagnostics14100984	Critical Risk Assessment, Diagnosis, and Survival Analysis of Breast Cancer	Preprocessing is important in enabling AI-enhanced individualized approaches to the management and treatment of breast cancer.
AI tools for clinical use	Lohaj et al., 10.3390/diagnostics14090917	Conceptually Funded Usability Evaluation of an Application for Leveraging Descriptive Data Analysis Models for Cardiovascular Research	Software usability should be evaluated in different dimensions, and can be improved through measures like a user manual and clear error messages for efficient feedback
AI tools for clinical use	Badahman et al., 10.3390/diagnostics14171870	Validating the Accuracy of a Patient-Facing Clinical Decision Support System in Predicting Lumbar Disc Herniation: Diagnostic Accuracy Study	AI-enhanced CDSSs provide a reasonable level of efficacy and may largely reduce the time and cost of screening of patients with lumbar disk herniation.
Summarization of machine learning models	Pinton, 10.3390/diagnostics14131324	Machine Learning for Predicting Biologic Agent Efficacy in Ulcerative Colitis: An Analysis for Generalizability and Combination with Computational Models	Machine learning models based on multiple pathways, multiple ethnicities, and real-world and clinical trial data are needed for data-driven decision-making and precision medicine. Data quality and quantity, overfitting, generalization, and interpretability are all unmet challenges.
